# The Effects of Interferons on Allogeneic T Cell Response in GVHD: The Multifaced Biology and Epigenetic Regulations

**DOI:** 10.3389/fimmu.2021.717540

**Published:** 2021-07-08

**Authors:** Chenchen Zhao, Yi Zhang, Hong Zheng

**Affiliations:** ^1^ Penn State Cancer Institute, Penn State University College of Medicine, Hershey, PA, United States; ^2^ Fels Institute for Cancer Research and Molecular Biology, Temple University, Philadelphia, PA, United States

**Keywords:** type I interferon, IFN- γ, GVHD, epigenetic regulation, alloreactive T cells

## Abstract

Allogeneic hematopoietic stem cell transplantation (allo-HSCT) is a potentially curative therapy for hematological malignancies. This beneficial effect is derived mainly from graft-versus-leukemia (GVL) effects mediated by alloreactive T cells. However, these alloreactive T cells can also induce graft-versus-host disease (GVHD), a life-threatening complication after allo-HSCT. Significant progress has been made in the dissociation of GVL effects from GVHD by modulating alloreactive T cell immunity. However, many factors may influence alloreactive T cell responses in the host undergoing allo-HSCT, including the interaction of alloreactive T cells with both donor and recipient hematopoietic cells and host non-hematopoietic tissues, cytokines, chemokines and inflammatory mediators. Interferons (IFNs), including type I IFNs and IFN-γ, primarily produced by monocytes, dendritic cells and T cells, play essential roles in regulating alloreactive T cell differentiation and function. Many studies have shown pleiotropic effects of IFNs on allogeneic T cell responses during GVH reaction. Epigenetic mechanisms, such as DNA methylation and histone modifications, are important to regulate IFNs’ production and function during GVHD. In this review, we discuss recent findings from preclinical models and clinical studies that characterize T cell responses regulated by IFNs and epigenetic mechanisms, and further discuss pharmacological approaches that modulate epigenetic effects in the setting of allo-HSCT.

## Introduction

Allogeneic hematopoietic stem cell transplantation (allo-HSCT) provides the long-term effective and curative treatment for patients with hematological malignancies. The therapeutic benefit of allo-HSCT is primarily attributed to the graft-versus-leukemia (GVL) effect, which is mainly mediated by infused donor T cells (1). However, these allogeneic T cells can also cause harmful graft-versus-host disease (GVHD) (2–4). Acute GVHD is a major risk for non-relapse mortality in the first 200 days after allo-HSCT (5). Therefore, maintaining the beneficial GVL effect while reducing GVHD is the holy grail of allo-HSCT.

Upon stimulation by host antigen-presenting cells (APC), infused donor T cells are activated to undergo robust proliferation and effector differentiation (2, 6). These APCs express high levels of antigen-presenting molecule MHC class II and costimulatory molecules (e.g., CD80, CD86), which are required to activate allogeneic T cells and promote expansion of activated T cells, respectively. Many cytokines, such as IL-2 and IL-12, are important for instructing these activated T cells to differentiate into effector cells mediating host tissue injury (7, 8). Notably, interferons (IFN) have an essential role in regulating T-cell activities during GVHD (9, 10). Type I (mainly IFN-α/β) and type II (IFN-γ) are two major IFNs that mediate pathophysiologic changes during infection, cancer and autoimmune diseases (11–15). IFN-γ is primarily derived from T helper 1 (Th1) CD4^+^ T cells and cytotoxic CD8^+^ T cells once adaptive immunity develops, whereas IFN-α can be produced by plasmacytoid dendritic cells (pDCs) (16, 17). Both IFN-γ and IFN-α are pivotal regulators of alloreactive T cell responses that mediate GVHD (18–20). However, optimal control of GVHD by modulating IFN signaling remains challenging. IFN signaling is complex and frequently context-dependent: it can lead to distinct effects at different times or stages of a disease course. IFNs regulate T cell functions by regulating a group of intracellular transcription programs. Epigenetic regulations of molecules in the IFN signaling pathway and the interferon-stimulated genes (ISG) are crucial for T cell activity (21, 22). This review focuses on how IFNs regulate alloreactive T cell responses and what role epigenetic regulation plays in this process.

## Effects of IFNs on T Cell Differentiation and Function During GVHD

### Type I IFNs

**Figure 1:**
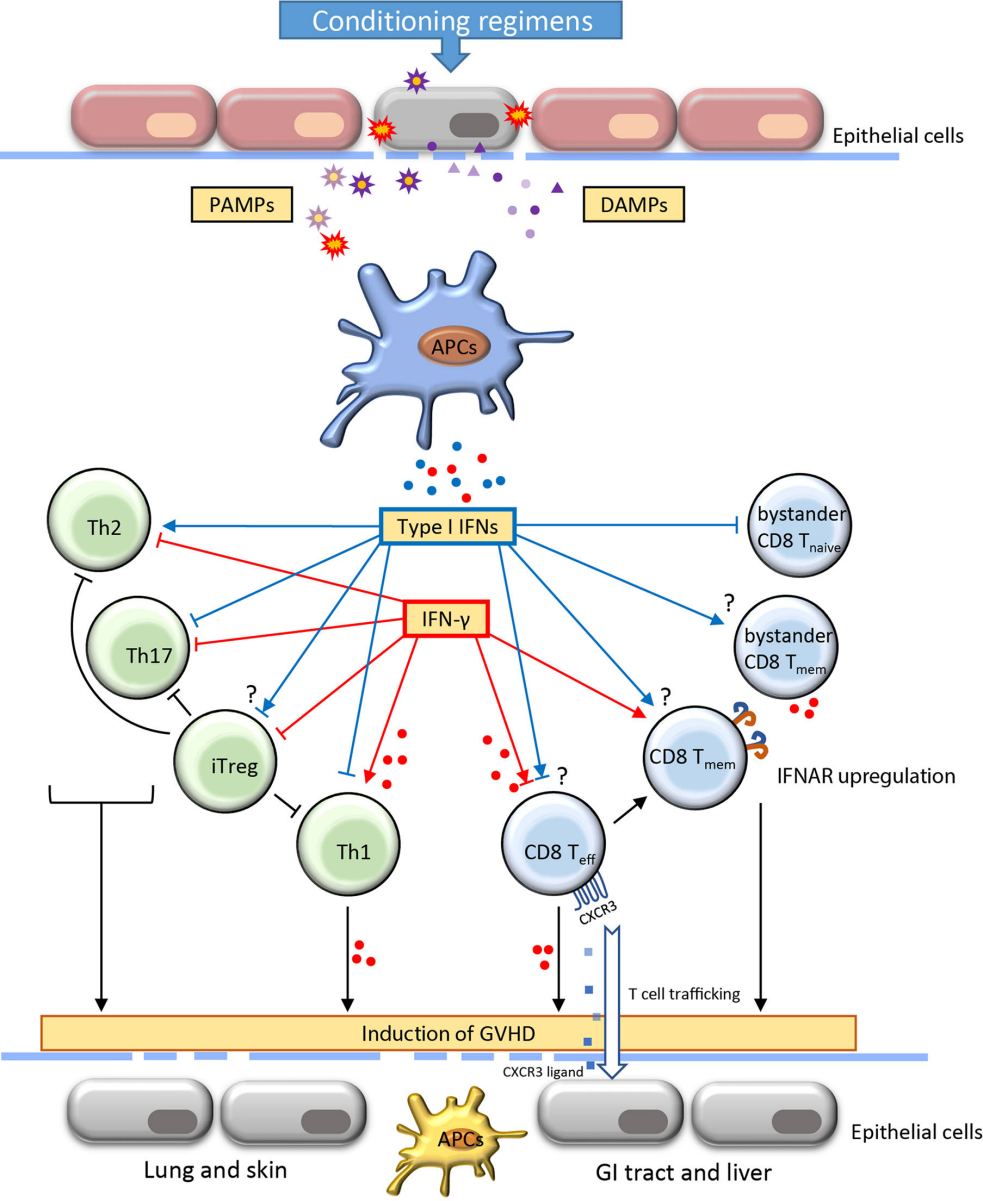
Role of type I IFNs and IFN-γ in the development of acute graft-*versus*-host-disease. Acute graft-*versus*-host disease (GVHD) is often initiated with the destruction of the epithelial barrier through the conditioning regimens including irradiation and chemotherapy. The signal of pathogen-associated molecular patterns (PAMP) and damage-associated molecular patterns (DAMP) released from damaged cells and microbiota induce the activation of antigen-presenting cells (APCs). Consequently, the production of IFNs by APCs interact with the alloreactive T cells and regulate their activation, differentiation, function and contraction. The proliferation of Th1 cells and effector CD8 T cells result in increased secretion of IFN-γ. The induction of activated alloreactive T cells and cytokines further affect the resident APCs and host tissues contributing to extensive functional incapability and damages of different organs. The IFNs play a critical role in orchestrating T cell activities throughout the induction and effector phase of GVHD. The blue and red dots indicate the IFNs (type I IFNs and IFN-γ, respectively) secreted by adjacent cells. CXCR, CXC chemokine receptors; GI, gastrointestinal; Th, T helper cells; iTreg, induced regulatory T cells; T_eff_, effector T cell; T_eff_, memory T cell.

Type I IFNs contain a subgroup of highly related polypeptides that have proven essential in regulating innate and adaptive immunity (23). Approximately 12-14 types of IFN-α and one type of IFN-β, IFN-ϵ, IFN-κ, and IFN-ω have been identified (24). Intriguingly, although type I IFNs are structurally divergent, only one form of heterodimer receptor, IFNAR, has been found. Thus, all type I IFNs activate the same receptor and many subsequent cell-signaling activities are shared. IFN-α and IFN-β are well defined and are the main subtypes from the immunological perspective. Virtually all cell types reserve the ability to produce variable level of IFN-β, whereas pDCs are the main source of IFN-α (23). Host tissue injuries triggered by conditioning regimens, such as preparative irradiation and chemotherapy, induce damage-associated molecular patterns (DAMP) and foreign pathogen-associated molecular patterns (PAMP). Type I IFNs are among the early cytokines whose production is triggered by the host and donor APCs after the detection of these danger signals by pattern recognition receptors (PRRs), such as toll-like receptors (TLRs) and nucleic acid sensors that located on or within the cytosol of cells (25) (**Figure 1**). IFN-α/β can exert antiviral and antitumor activity by up-regulating MHC-I and subsequently promoting antigen presentations.

IFN-α/β has context-dependent roles in CD4^+^ T cell activation, differentiation and survival (26). IFN-α is associated with CD4^+^ T cell activation and contributes to the IFN-γ-mediated Th1 response (27). In contrast, IFN-α/β may suppress Th2 differentiation of human CD4^+^ T cells. Importantly, IFN-α regulation of T cell differentiation appears to be context-dependent. Dichotomous T cell polarization towards either Th1 or T_FH_ was recently observed depending on the IFN-α exposure at different times (28, 29). In a colon-targeted GVHD murine model, IFN-α signaling prevented donor CD4^+^ T cell proliferation and differentiation, resulting in alleviating colon tissue damage (30, 31). Regulatory T cells (Tregs) play essential roles in controlling immune tolerance after allo-HSCT (32). Adoptive transfer of Treg ameliorates GVHD and improves survival in a murine model (33). However, IFN-α/β has shown some controversial impacts on Tregs. Some studies suggested that overexpression of IFN-α significantly reduced the frequency of Tregs in the tolerogenic tumor environment (34). Other studies suggested that IFN-α stimulation may increase differentiation of CD4^+^CD25^+^FOXP3^+^ Tregs (iTregs) (35) (**Figure 1**).

IFN-α/β signaling is essential for antigen-driven CD8^+^ T cell responses. First, differentiation of effector CD8^+^ T cell was associated with decreased IFNAR but increased IL-12 receptor, whereas augmented IFNAR favors the development of central memory T (T_CM_) cell (36, 37). In IFNAR deficient mice, CD8^+^ T cells lose the ability to become memory T cells during lymphocytic choriomeningitis virus (LCMV) infection (37, 38). Paradoxically, withdrawal of IFN-α monotherapy in clinical chronic myeloid leukemia resulted in elevated frequency of peripheral CD8^+^ T_CM_ cells (39). Given that many different types of cells express IFNAR, the difference in regulating memory formation between IFNAR deficiency and IFN-α monotherapy may be attributable to both direct and indirect mechanisms. Second, the activation of IFN-α/β signaling in T cells could benefit cytokine secretion and cytolytic activity. In mice, injection of IFN-α incited substantial primary CD8^+^ T responses through cross-priming by DCs that were independent of CD4^+^ T-cell help (40, 41). IFN-α/β signaling plays a co-stimulatory role in CD8^+^ T activation and slows the death of activated T cells (42, 43). Moreover, direct activation of granzyme B transcription through IFN-α/β in effector CD8^+^ T cells contributes to tumor suppression as well as autoimmunity (44, 45). Consistent results were found in the context of GVHD that both CD8-dependent GVHD and GVL effects were enhanced through IFN-α/β signaling (30). In addition, despite IFN-α/β signaling induces transient attrition of bystander naïve T cells in the wake of T-cell response, it can rapidly activate nonspecific bystander memory CD8^+^ T cells. Activation of memory T cells contributes to rapid production of proinflammatory cytokines including IFN-γ (**Figure 1**) (46–48).

Clinically, recombinant IFN-α has been used alone or in combination with donor lymphocytes infusions or other cytokines such as granulocyte-macrophage colony-stimulating factor to establish GVL effects in patients with minimal residual disease or relapse after allo-HSCT (49–53). Studies using animal models demonstrate the effectiveness of IFN-α treatment against leukemia cells. A most recent trial showed that the proportion of granzyme positive CD8^+^ effector and effector memory subsets is positively correlated with GVHD incidence (53). This suggests that IFN-α-induced CD8^+^ T cells may be a double-edged sword against both malignant and normal cells.

In response to IFN-α/β signaling, the trimolecular interferon-stimulated gene factor 3 (ISGF3), which comprises signal transducer and activator of transcription (STAT) 1, STAT2 and interferon regulatory factor (IRF) 9, leads to most of the cellular effects of IFN-α/β (23). The dysregulation of ISGF3 results in aberrant T cell functions. For example, the absence of IRF2, a negative regulator of ISGF3, induces hyperresponsiveness of CD8^+^ T cells and promotes spontaneous inflammatory skin lesion in mice (54). Furthermore, in a LCMV infection mouse model, the STAT1 deficiency leads to a CD4^+^ T cell-mediated lethal disease. This effect is independent of IFN-γ, but it coincides with exaggerated proinflammatory cytokine production as well as increased frequency of LCMV-specific CD4^+^ and CD8^+^ T cells (55). These observations suggest that the effects of IFN-α/β signaling are not only divergent on CD4^+^ or CD8^+^ T cells, but also highly dependent on the pathophysiological backgrounds. Of note, several molecules in the downstream of the IFN-α/β pathway, including Janus Kinase (JAK) 1, STAT1 and STAT3, are shared with IFN-γ signaling, which indicates the possibility of crosstalk between the two signals in the transcriptional level.

### IFN-γ

IFN-γ promotes CD4^+^ T cell differentiation towards Th1 lymphocytes and drives CD8^+^ T cell expansion and differentiation towards both effector and memory cells (**Figure 1**). Early studies in GVHD models suggest that IFN-γ contributes as a pathogenic factor to alloreactive responses. For instance, high serum levels of IFN-γ correlated with increased severity of GVHD after allo-HSCT (56). IFN-γ induced apoptosis of intestinal epithelial crypt cells, leading to extensive erosion of intestinal epithelium and GVHD propagation (57, 58). Genetic deletion of IFNGR in T cells prevents lethal GVHD while preserving the robust GVL effect (59). Furthermore, evidence from live-cell imaging reveals that both motility and cytotoxicity of CD8^+^ T cells are enhanced in alloreactive tissue due to autocrine/paracrine IFN-γ (60). The expression of CXCR3 induced by IFN-γ signaling is one of the mechanisms that drive the T cells to the sites of GVHD target organs (59).

Intriguingly, some studies suggested that IFN-γ may negatively regulate alloreactive T cells and prevent tissue damages. Evidence from IFN-γ knockout mice shows that IFN-γ could be protective against GVHD depending on the extent of conditioning in mouse models (18, 61). Infusion of IFN-γ-null donor T cells increased mortality of GVHD compared to that of wild-type T cells (62). One possible reason might be that IFN-γ is required for normal T cell contraction since IFN-γ deficiency would lead to delayed apoptosis of CD8^+^ T cell population, leading to prolonged inflammation (63–65). In addition, PD-L1, which is considered as an inhibitory checkpoint molecule in infections and tumors, was identified as a positive contributor to T cell-mediated GVHD in the murine model, as decreased inflammatory cytokines and increased apoptosis were observed in both *Pdl1* ^-/-^ allogeneic CD4^+^ and CD8^+^ T cells. Of note, both *Ifngr^-/-^* CD4^+^ and CD8^+^ donor T cells showed impaired PD-L1 expression, suggesting that loss of IFN-γ signaling mitigates tissue damages in GVHD *via* the PD-L1 pathway (66).

Interestingly, manipulation of IFN-γ signaling in alloreactive T cells results in variable lesions in GVHD target organs. IFN-γ produced by alloreactive T cells is the primary mediator contributing to the apoptosis of intestinal stem cells and intestinal damage (57). In addition, both clinical and preclinical studies suggest that IFN-γ-producing Th1 cells mediate damages in the gastrointestinal (GI) tract (67), whereas IFN-γ KO model results in exacerbated skin and lung injury (68). In the absence of IFN-γ signaling, alloantigen-primed CD4^+^ T cells showed decreased capacity to produce IFN-γ-secreting Th1 cells while skewing toward both Th2 and Th17 cells (68). Further studies are needed to define the correlation between the effects of IFN-γ on alloreactive T cells and the consequence of GVHD.

**Figure 2:**
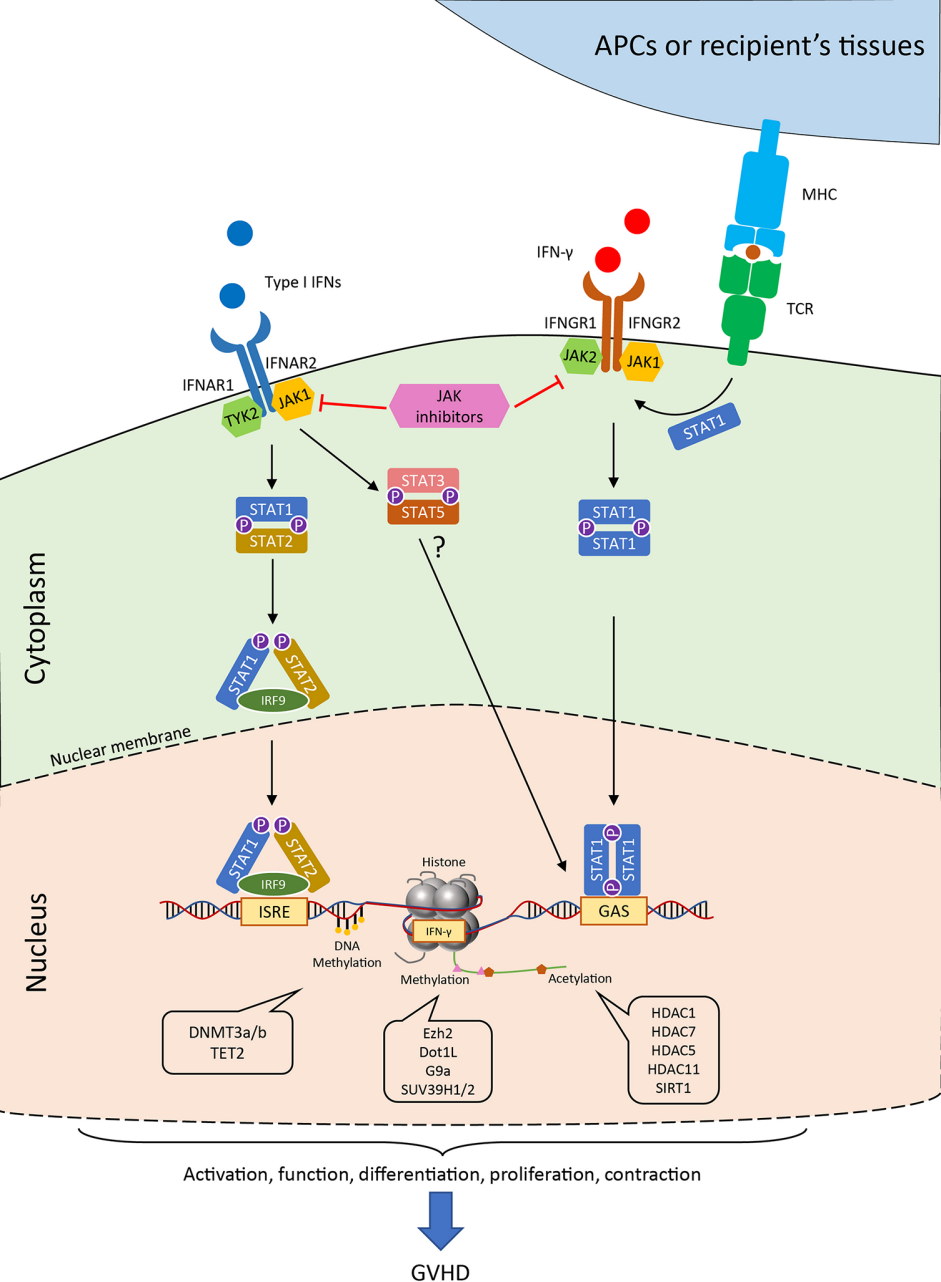
Janus kinase-signal transducer and activator of transcription (JAK-STAT) pathways and the epigenetic regulations of IFN signaling in T cell. Generally, the binding of type I IFN to the receptor initiates the engagement of IFNAR1 associated Tyk2 protein tyrosine kinase and the IFNAR2 associated JAK1 protein tyrosine kinase. The signal further passes to the phosphorylation and the heterodimerize of STAT1 and STAT2, which together with IRF9 form the ISGF3 complex in the cytoplasm. ISGF3 translocates into the nucleus and binds to IFN-stimulated response elements (ISREs) found in most of IFN-stimulated genes (ISGs). Alternatively, STAT3 and STAT5 heterodimer are also observed after IFNAR activation in the absence of STAT1. Canonical IFN-γ signaling occurs through IFNGR and activates the JAK1/2 kinases, which further induce the phosphorylation of STAT1. The STAT1 homodimer can directly move into the nucleus and binds to gamma-activated sequence (GAS) sites. The activation of T-cell receptor could also help the co-localization of STAT1 to IFNGR-rich regions of the membrane. The transcription of the downstream genes as well as the production of IFN-γ are tightly regulated by numerous epigenetic enzymes, which control the modification of the DNA and histones. These regulators critically control the T cell activities in the process of GVHD, which allows for possible therapeutic interventions. JAK, Janus kinase; STAT, signal transducer and activator of transcription; IFNAR, interferon alpha receptor; IFNGR, interferon gamma receptor; ISREs, IFN-stimulated response elements; GAS, gamma-activated sequence; TCR, T cell receptor; MHC, major histocompatibility complex; IRF, interferon regulatory factor; HDAC, histone deacetylase; SIRT, sirtuin.

The binding of IFN-γ to the receptor, IFNGR1 and IFNGR2 complex, induces recruitment and phosphorylation of receptor-associated JAK1/2, which triggers subsequent signaling pathways predominantly through STAT1 (**Figure 2**). Interestingly, TCR stimuli initiate the translocation of STAT to IFNGR1-rich regions of the membrane similar to IFN-γ ligation (69). Blocking the JAK1/2 molecule significantly abrogates the polarization and proliferation of activated T cells as well as downregulates activation markers, such as CD69 and CD25, and reduces the production of proinflammatory cytokines (70). In light of the suppressive effect on T cell responses, the JAK inhibitors were reported to control GVHD in both mice and humans. Recently, ruxolitinib was approved for the treatment of steroid-refractory acute GVHD. JAK inhibitors mitigate GVHD *via* pleiotropic effects on T cells. For example, ruxolitinib mitigates acute GVHD by reducing CXCR3 expression, which results in less T-cell infiltrates in target organs (59, 71, 72), and by decreasing IFN-γ and IL17A production in CD4^+^ T cells (73). Similarly, another JAK1/2 selective inhibitor, baricitinib, can abrogate IFN-γ and IL-6 signaling in CD4^+^ T cells and significantly decrease Th1 and Th2 cell differentiation while augmenting the frequency of Tregs (74). In addition to the reduction of GVHD, baricitinib could also improve GVL (74). Despite these promising observations, the transcriptional regulations of the downstream genes in T cells are yet to be found.

Notably, as the IFNGR can be expressed on almost all cell types, the generated IFN-γ from activated allogeneic T cells could have a remarkable influence on the innate immunity and break the homeostasis of the surrounding tissues. For instance, IFN-γ signaling acts as a ‘super-activator’ of macrophages, inducing transcriptional activation of proinflammatory genes (e.g., IL-6 and TNF-α) and enhancing antigen presentation. Furthermore, recent evidence suggests that IFN-γ directly inhibits the proliferation of hematopoietic stem/progenitor cells (HSPCs) and their generation of pDCs that can induce immune tolerance against GVHD (20). On the other hand, exposure to IFN-γ reduces the proliferation of intestinal epithelial cells and further induces apoptosis by modulation of the AKT/β-catenin and Wnt/β-catenin pathway (75). Thus, although IFN-γ plays dichotomous roles in the regulation of T cells, its proinflammatory damage to the tissues in GVHD is generally acknowledged (**Figure 1**).

### Type III IFNs

Recent studies have discovered other members of type III IFNs, including IFN-λ1 (IL-29), IFN-λ2 (IL28A), IFN-λ3 (IL-28B) and IFN-λ4 (76–78). They participate in the antiviral activities similar to the type I IFNs, but primarily in barrier tissues such as mucosal epithelial cells (79, 80). Although IFN-α/β and IFN-λ engage different receptors, they induce similar downstream signaling pathways through the phosphorylation of STAT1/2 and the subsequent transcription factor ISGF3 (81). However, our knowledge of IFN-λ in GVHD is limited. An initial study found that IFN-λ2 did not significantly modulate GVHD mortality in a murine model upon deleting its receptor (*Ifnlr1^-/-^*) or administration of recombinant IFN-λ2 (82). Intriguingly, a most recent study revealed that *Ifnlr1* deletion led to exaggerated damages in the GI tract and recombinant IFN-λ treatment reduced GVHD lethality (83). Deletion of *Ifnlr1* led to increases of donor T-cell expansion and serum IFN-*γ* levels, however, it did not affect the proliferation and apoptosis of alloreactive T cells. Interestingly, the effect of IFN-λ on T cells seemed to be indirect since the T-cell expansion was influenced by early engraftment, which was related to IFN-λ signaling in NK cells (83). Further studies of IFN-λ in the modulation of GVHD the underlying mechanisms are warranted.

## Epigenetic Regulation of IFN Expression and Function in T Cells

The epigenomic signatures, including DNA methylation on cytosine nucleotides, histone modifications and chromatin accessibility, reflect previous and present gene expression, and can positively or negatively regulate future transcription according to environmental stimuli. The labeled or ‘bookmarked’ chromatin organized as ‘epigenetic code’ that can be recognized by protein complexes called ‘readers’. It is also closely controlled by enzymes, called ‘writers and erasers’, that are able to manipulate different modifications mounted on specific residues (84). The major contributors comprise DNA methyltransferases (DNMTs) or DNA demethylases on DNA level; histone acetyltransferase (HATs), histone deacetylase (HDACs), histone methyltransferases (HMTs) and lysine demethylases (KDM) on histone level. Other epigenetic mechanism includes the microRNAs (miRNAs), which negatively control target gene expression post-transcriptionally *via* interaction with the complementary sequences. It has been well established that IFN-signaling can generate ‘interferon epigenomic signatures’ and reprogram cell response (21).

During antigen-driven immune responses, such as GVHD, T cells are located at the downstream of type I IFN signaling as they receive this signal from innate immune cells (20, 85). Although many regulators and pathways of type I IFN signaling from innate cells may be interchangeable in T cells, the cell type and context-dependent mechanisms have yet to be characterized. As discussed earlier, the canonical signal of type I IFN depends on the activation of STAT1 and STAT2, which leads to the activation of ISGs by transcription factor IRF9 (also known as ISGF3) (23). Notably, multiple pathways co-exist in the downstream of IFNAR1/2 and control T cell immune responses (**Figure 2**). For example, IFN-α/β induced STAT1 is responsible for the suppression of CD8^+^ T cell expansion, whereas STAT3 and STAT5 mediate antiapoptotic and mitogenic effects in T cells in the absence of STAT1 (86).

The epigenetic regulation of ISGs in T cells is far less documented compared with studies in innate immune cells. Evidence has been widely found on innate immune cells that ISG promoters are associated with increased level of histone acetylation, which in part mediated by STAT1 and STAT2 (87), STAT1/2 promotes histone acetylation after IFN-α/β stimulation in T cells as well. Early study has linked IFN-α signaling with histone hyperacetylation at the granzyme B and eomesodermin (Eomes) loci during CD8^+^ T cell differentiation (88). Similarly, IFN-α/β signaling enhances H3K4me3 and H3K9ac (transcription permissive) at the promoter region of Eomes and activates it in an IRF9-dependent manner (89). Interestingly, T-bet is found to counteract aberrant IFN-α/β signaling during Th1 cell development by repressing ISGs such as *Isg15*, *Mx1*, Oasl1a, etc. Deletion of T-bet results in accumulation of STAT2 and elevation of transcription active mark H3K27ac at ISGs activated by IFN-β (90), highlighting the complexity of interactions between the extrinsic cytokine influence and the intrinsic regulation of cell development. In addition, the epigenetic modulation of T cells in responding to IFN-α/β is likely to be context-dependent. For example, although STAT1 mRNA levels are both increased in lupus and normal CD8^+^ T cells with IFN-α stimulation, the signature of hypomethylated DNA sites in lupus CD8^+^ T cells facilitates the upregulation of HLA-DRB1 in a STAT1-signaling-dependent manner (91). In addition to histone and DNA modification, the miRNA-155 downregulates the T-cell responsiveness to IFN-α/β *via* IFNAR-STAT pathway. Despite the direct targets of miRNA-155 were not defined, the loss of microRNA-155 results in impaired antiviral CD8 T cell response (92). To reconcile these paradoxical and the highly context-dependent effects of IFN-α/β on T cells, it will be important to map how the ISGs are regulated on the epigenetic level in alloreactive T cells during GVHD.

Compared to IFN-α/β, the epigenetic regulation of IFN-γ is much more complicated. IFN-γ not only promotes antigen-driven T cell differentiation, but also is the major mediator for tissue injury. Much work has investigated the epigenetic regulation of *Ifng* locus in T cells. The CpG dinucleotide at *Ifng* promoter in naïve CD8^+^ T cells is substantially methylated and undergoes demethylation when these CD8^+^ T cells are activated. Memory CD8^+^ T cells retain relative hypomethylated status to enable a rapid gene expression for the re-activation in the future (93, 94). Similar regulation can be found in Th1 CD4^+^ T cells. The CpG for *Ifng* promoter in naïve CD4^+^ T cells is mostly methylated, but only approximately 50% of CpG regions are methylated in resting memory cells (95). Conversely, in the lineages other than Th1 development, their suppression of IFN-γ can also be achieved in part with DNA methylation (96). The ten-eleven translocation (TET) 2 enzyme, which mediates DNA demethylation, positively regulates *Ifng* transcription by promoting 5-hydroxymethylcytosine level in CD4^+^ T cells (97). From another view, histone modifications act in accordance with the gene regulation of DNA methylation. For example, the *Ifng* promoter region in Th1 cells is associated with hyperacetylation of histones H3 and H4, but not in Th2 cells (98). Additionally, the *Ifng* suppression in Th2 cells is accompanied by repressive H3K9me3, which is governed by enzyme SUV39H1. Loss of this enzyme results in skewed lineage stability (99).

## Epigenetic Programs and Pharmacological Modulations That Control IFNs in Allogeneic T Cells During GVHD

The function and differentiation of T cells are closely intertwined with IFN expression. Epigenetic processes, including DNA methylation, histone modification and chromatin remodeling, are the key mechanisms that control T cell differentiation and function (100, 101). Multiple epigenetic enzymes have been identified to regulate the production and subsequent effect of IFNs in allogeneic T cells (102–104). A number of chemical compounds that selectively inhibit these enzymes are made available, and their effect on IFN signaling and therapeutic potentials are under active investigation (104–106). Since there are very limited reports studying the epigenetic regulation of type I IFNs in the GVHD context, our continual discussion will focus on the epigenetic effects on IFN-γ.

### DNA Methylation

DNA methyltransferase (DNMT) 3a or DNMT3b contributes to *de novo* DNA methylation, resulting in genetic silencing. In T cells, DNMT3a expression is regulated by TCR signaling (107). The promoter of *Ifng* locus remains hypomethylated during Th1 differentiation from naïve CD4^+^ T cells, albeit *de novo* DNA methylation at *Ifng* promoter is observed in other commitments, such as Th2, Th17 and iTreg cells (108). DNMT3a is responsible for maintaining the silence of *Ifng* gene in non-Th1 lineages (109). Accordingly, deletion of DNMT3a after T cell activation selectively reduces the level of *Ifng* methylation (107), and allows significant IFN-γ production from non-IFN-γ producing CD4^+^ T cells (109). During secondary contact with antigen, DNA demethylation at the IFN-γ promoter takes place in memory T cells in order to facilitate rapid effector responses (94). Furthermore, the functional exhaustion of the CD8^+^ T cells couples with persistent DNA hypermethylation at *Ifng* loci, even if the cells are treated with anti-PD-1 blockade. Inhibition of DNA methylation by hypomethylating agent together with anti-PD-L1 significantly promotes IFN-γ secretion by exhausted CD8^+^ T cells (110). The above studies suggest that inhibition of DNMT may promote alloreactive T cell activities in GVHD by suppressing DNA methylation and subsequently enhance IFN-γ production.

Although hypomethylating agents globally alter DNA methylation levels, their influence on gene expression in T cells shows preference. Compelling evidence indicates that both IFN-γ and FOXP3 locus are demethylated by Azacytidine (Aza) (94, 111, 112). In vitro studies revealed that Aza and decitabine could directly induce FOXP3 expression in T cells, whereas IFN-γ gene expression along with other cell-cycle related genes were significantly down-regulated by Aza (104). Consistently, decitabine significantly suppressed differentiation of naïve CD4^+^ T cells into Th1 subsets but not Tregs (113). These findings applied to the alloreactive T cells in GVHD. It has been observed that Aza mitigates GVHD in murine models by converting alloreactive CD4^+^CD25^+^FOXP3^-^ cells to suppressive CD4^+^CD25^+^FOXP3^+^ Tregs and directly increase Treg proliferation. In addition, the frequency of IFN-γ-producing CD4^+^ T cells was significantly decreased (114–116). The inhibition of naïve CD4^+^ T cell proliferation by decitabine is also accompanied by the elevation of the TET2, an enzyme that acts opposite to DNMTs, which promote DNA demethylation (113).

Despite the above *in vitro* data supporting that hypomethylating agents up-regulate Treg and suppress conventional CD4^+^ T cells (117, 118), post-transplantation Aza treatment in patients with high risk of AML and MDS shows no significant differences in terms of overall survival and GVHD incidence in patients compared to the control arm (119). It is possible that additional epigenetic mechanisms are involved in the IFN-γ regulation of alloreactive T cells. For example, in DNMT3a-null Th2 or Th17 cells, decreased level of DNA methylation at the *Ifng* loci correlated with low level of H3K4 and high H3K27 methylation, which permits and inhibits DNA transcription, respectively (109).

### Histone Methylation

Histone methylation is predominantly restricted to the N-terminal tails of H3 and H4 histones and is usually presented by one, two, or three lysine residues (120). The effects of histone methylation on gene expression are loci-specific. Genes that bound by H3K4, H3K36 and H4K20 are more likely to be actively transcribed, whereas H3K9, H3K27 and H3K79 are usually associated with gene suppression (121–124). The histone methylation level at each site is controlled by one or a set of HMTs and KDMs (120, 124). Thus, the activities of these enzymes are the key factors that determine gene transcription.

CD4^+^ and CD8^+^ T cells display unique patterns of histone methylation landscapes at *Ifng* locus based on the stages of cell differentiation. Once activated by TCR signaling or specific cytokines, the histone methylation markers of T cells are dynamically catalyzed by their dedicated enzymes. During the quiescent stage of naïve T cells, the *Ifng* promoter of both CD4^+^ and CD8^+^ T cells are occupied with repressive H3K27me3 but low level of permissive H3K4me3 (125, 126). Upon activation, *Ifng* region of both CD4^+^ and CD8^+^ T cells loses H3K27me3 markers (125, 127, 128). However, effector CD8^+^ T cells gain H3K4me3 at *Ifng* locus (127, 129). CD4^+^ Th1 cell differentiation increases both H3K4me3 and H3K9me2 (permissive and repressive, respectively), whereas CD4^+^ Th2-cells rapidly extinguish H3K9 methylation by STAT6 and GATA-3 dependent mechanisms (130).

Ezh2 is a crucial enzyme that catalyzes H3K27 methylation and remarkably silences target genes to facilitate T cell differentiation and function. During Th2 cell development, Ezh2 is recruited with STAT6 and GATA3 to the *Ifng* locus and is responsible for the silencing of *Ifng* locus through H3K27 methylation (130). *In vitro* studies revealed that Ezh2 affected CD4^+^ T cell differentiation depending on the context of the extracellular environment. For instance, Ezh2-deficiency could enhance the CD4^+^ T cell production of either IFN-γ or IL-4, depending on the cell-inducing cytokines *in vitro*, such as IL-12 or IL-4, respectively (131–133). Further, both T-bet and Eomes are required for the regulation of IFN-γ production by Ezh2 (131). The role of Ezh2 in GVHD is complex. In an MHC-mismatched B6 anti-BALB/c GVHD murine model, loss of Ezh2 in donor T cells resulted in impaired IFN-γ production and reduced GVHD. Specifically, Ezh2 promoted Th1 development by stimulating *Ifng*, *Tbx21* and *Stat4* expression (102). Similar results could also be found from Th1 cells in aplastic anemia, in which Ezh2 directly activated *Tbx21* transcription by direct binding to its promoter (134). Contradictive results are also found with CD8^+^ T cells. Ezh2 inhibition resulted in increased frequency of IFN-γ producing tumor-infiltrating CD8^+^ T cells (135), whereas Ezh2-deficient CD8^+^ T cells exhibit an impaired ability to produce IFN-γ in a virus infection model (136). How to explain the discrepancy observed in these studies remains elusive. In addition, Ezh2 could co-localize with FOXP3 and assist in silencing the IFN-γ expression (137). Consistently, the absence of Ezh2 resulted in defective Treg differentiation, which could further contribute to autoimmune colitis (132).

Dot1L, a solo H3K79 methyltransferase, has been recently identified to regulate T cell activation and polarization. In general, H3K79 methylation strongly correlates with active gene transcription (138, 139), but exceptions are also reported (140, 141). When T cells were cultured in Th1 cell-polarizing conditions, IFN-γ production was enhanced by Dot1L inhibition with a small molecule inhibitor (SGC0946) at the beginning of polarization and was associated with the reduction of H3K79me2. Interestingly, the proliferative capacity was not affected (142). These observations indicate that Dot1L may play a negative role in regulating Th1 cell differentiation and IFN-γ production. Another group recently used a T-cell-specific Dot1L-deficient infection mouse model and observed that the repressive effect of IFN-γ production by Dot1L was T-bet dependent. In this study, the enhanced IFN-γ secreting ability *via* Dot1L inhibition (with chemical probe SGC0946) in Th2 cells was abrogated by T-bet deletion (143). However, the opposite phenomenon was observed in GVHD setting. Inhibition of Dot1L with the same chemical probe attenuated xenogeneic GVHD by globally suppressing T cell activation-induced genes, in which IFN-γ production was significantly reduced (103). Of note, this effect was only observed in T cells with low-avidity TCR interaction. Therefore, Dot1L inhibition increased the TCR stimulation threshold and was controlled in an ERK phosphorylation-dependent manner (103). The inconsistent findings among different studies are likely due to the different roles of Dot1L in the regulations of upstream and downstream of IFN-γ signaling. Similar to the data found in CD4^+^ T cells, Dot1L also remarkably controls the differentiation of CD8^+^ T cells. Dot1L-deficiency resulted in the induction of memory-like transcriptome feature in antigen inexperienced CD8^+^ T cells. Furthermore, these cells were functionally impaired as they were incompetent to produce IFN-γ upon stimulation with anti-CD3 and anti-CD28 antibodies (144). In addition, using the approach of genetic Dot1L deletion and a specific inhibitor, EPZ004777, the repression of Dot1L resulted in inhibition of H3K79me2 in CD8^+^ T cells that associated with increased CD8^+^ T cell apoptosis and suppressed IFN-γ and TNF-α secretion. Besides, the methionine metabolism in the microenvironment also affects the methylation status of H3K79 in CD8^+^ T cells, further promoting the dysregulation of the immune response (145). However, genetic approaches are required to define the precise role of Dot1l in T cells.

Both G9a and SUV39H1/2 contribute to the methylation of the H3K9 site. However, G9a catalyzes H3K9 residue to mono- or dimethylation (H3K9me/me2), whereas SUV39H1/2 is responsible for di- to tri-methylation (H3K9me3) (146, 147). These enzymes could be found in multiple repressive complexes that promote transcription inhibition. Importantly, the heterochromatin protein 1α (HP1α) directly recognizes and binds to H3K9me3 and initiates the chromatin remodeling by forming heterochromatin (148). Despite their similarity in histone modification, G9a and SUV39H1/2 are remarkably divergent in epigenetic regulation of IFN and its subsequent effects on T cell functions. During Th2 development, G9a facilitates the transcriptional silence of *Ifng* locus since increased IFN-γ production was observed in G9a deficiency CD4^+^ T cells along with a decreased level of H3K9me2 (149). However, given that G9a deficiency and inhibition do not affect the development of Th1 cells as well as their capacity in secreting IFN-γ both *in vitro* and *in vivo*, G9a is currently considered dispensable for Th1 cell response (149, 150). Similarly, the ability to produce IFN-γ in CD8^+^ T cells is not affected in G9a knockout cells, but G9a is crucial to repress helper T lineage genes after the activation of CD8^+^ T cells (151). These studies indicate a moderate role of G9a in epigenetic control of IFN-γ. In addition, although not verified in T cells, evidence suggests that the downstream of IFN-α/β signaling and ISGs are negatively regulated by G9a (152). On the other hand, SUV39H1-H3K9me3-HP1α pathway also contributes to Th2 stability by decorating H3K9me3 at *Ifng* promoter (99). Less is known whether this signaling redundancy may mutually compensate for both G9a and SUV39H1 when activated *via* different upstream pathways.

In addition, SETDB1, which belongs to the SUV39H family, is responsible for H3K9me3 deposition at specific promoters. Adoue et al. demonstrated that SETDB1 was required to maintain IFN-γ silencing in Th2 cells. Instead of directly catalyzing H3K9me3 on the target gene, SETDB1 represses adjacent endogenous retrovirus location that affects the transcription of Th1 genes (153). This study reveals the spatial regulation of histone modification in the epigenetic control of Th cell differentiation. In contrast to the inhibitory regulation of IFN-γ in CD4^+^ T cells, wild-type CD8^+^ T cells exhibited a higher ability to produce IFN-γ compared to SUV39H1-conditional knockout mice that infected with *L. monocytogenes.* SUV39H1 was responsible for silencing stem/memory gene programs while enhancing the functions of effector cells in CD8^+^ T cells (154).

### Histone Acetylation

Unlike methylation, histone acetylation uniformly assists gene transcription because the acetyl group neutralizes the positive charge on the histones, thereby reducing the electrostatic force between histone and the negatively charged DNA molecules (155). On the other hand, together with methylation, phosphorylation and other covalent modifications, histone acetylation also takes part in the formation of ‘epigenetic code’, which allows the recognition by the protein complexes that help amplify the gene transcription (156). HAT and HDAC regulate acetylation status on both H3 and H4 histones.

The anti-inflammatory properties of the HDAC inhibitors have long been recognized by numerous experimental and clinical studies, including GVHD. Early studies that first linked histone acetylation with GVHD revealed that the panoramic HDAC inhibitor (pan-HDACi) suberoylanilide hydroxamic acid (SAHA) ameliorate and delayed the development of GVHD and reduced the serum level of proinflammatory cytokines such as IFN-γ and TNF-α following allogeneic bone marrow transplantation (157, 158). Although the STAT1 phosphorylation was inhibited during this process, the T cell proliferation and cytotoxic responses in GVL activity remained intact. Several clinical trials using SAHA as prophylactic treatment after allo-HSCT also observed reduction of GVHD in patients (159, 160). These trials revealed higher Treg cell numbers in the peripheral blood after HDACi administration as well as lower GVHD-related biomarkers, such as ST2 and Reg3α, and the proinflammatory cytokine IL-6 in the plasma. Another clinical trial uses pan-HDACi panobinostat, combined with glucocorticoids, as primary treatment for acute GVHD demonstrates an enhanced H3 acetylation in both CD4^+^ and CD8^+^ T cells (161). However, in contrast to the mouse model, the level of plasma IFN-*γ* was not significantly changed in patients (160, 161). It will be interesting to determine whether functional changes of T cells are controlled by HDACi and correlate with clinical responses and outcomes of patients.

Accumulating evidence from recent studies discovered the regulation of IFN-γ by specific HDAC members in T cell responses with or without allogeneic antigens. HDAC1-deficiency does not affect the development and effector functions but increases the STAT1 activity in CD4^+^ T cells, which results in the elevated level of IFN-γ production in activated Th1 cells (162, 163). Similar effects were also detected in effector CD8^+^ T cells (164), indicating a negative role of HDAC1 in controlling IFN-γ transcription. Besides, HDAC7 and SIRT1 may synergize with HDAC1 in repressing T cell activation and IFN-γ production *via* separate pathways (165, 166). On the contrary, HDAC5 and HDAC11 positively regulate IFN-γ production. Both HDAC5 deletion in CD8^+^ T cells and HDAC11 knockout in CD4^+^ and CD8^+^ T cells induce increased IFN-γ production upon anti-CD3 and anti-CD28 activation *in vitro* (167, 168). In addition, potent GVHD in the murine model can be induced in HDAC11 KO mice. Both CD4^+^ and CD8^+^ T cells with HDAC11 deletion are hyperresponsive to alloantigen and associated with increased expression of Eomes and T-bet (167).

Of note, inconsistent results can be found among studies. These discrepancies may partially attribute to the fact that some HDACs can both have histone and non-histone targets, which increases the complexity of gene regulation by introducing indirect effects. For example, genetic deletion and inhibition of SIRT1 reduces T-cell alloreactivity and promotes the function of iTreg through the enhancement of p53 acetylation, leading to the attenuation of GVHD (169).

Compared to the extensive investigations exploring the features of HDACs, the regulatory role of HAT in IFN related T cell responses are not well understood. In Th2 cells, the HAT p300 is recruited by Gata3 and Chd4 complex to promote the transcription of Th2 cytokine, whereas HDAC2 is recruited in the Gata3-Chd4-NuRD complex to suppress *Tbx21* and the subsequent IFN-γ expression (170). Moreover, the CREB-binding protein and p300 complex regulates the differentiation of human Treg *via* H3K27 acetylation. Although much evidence has been found in innate immune cells that p300 and other HATs essentially regulate STAT-ISG signaling and type I IFN production, our understanding of the epigenetic control of HATs remains low in regard to functional regulation of IFN in T cells. Especially, further studies of HATs should be conducted to validate the roles of both HATs and HDACs in GVHD models.

## Conclusion and Perspectives

Although extensive efforts have been made in defining the roles of IFNs in alloreactive T cells, the mechanisms underlying the effects of IFNs in the setting of GVHD remain largely unknown. Much of our knowledge about the IFN-related regulations in T cells mostly come from infection, tumor and autoimmune diseases. However, considering the release of DAMP and PAMP, anti-leukemia effect and the exposure of alloreactive antigens in patients after allo-HSCT, the T cell response in GVHD and GVL scenarios reflect combined effects of these conditions. In addition, the effect of IFNs in different organs and tissues, which have distinct microenvironments, may also affect T cell response. Development of novel genetic approaches is important to further dissect the impact of IFNs on T cell alloimmunity and tumor immunity.

The advancement in epigenetics of T cell biology opens a unique way to understand the molecular mechanisms of IFN regulation. Much effort has been made to identify key epigenetic enzymes and pathways that affect IFN expression in T cells. However, most of the effects of the enzymes are still unknown in the context of GVHD. It will be interesting to determine the connection between the inhibitors of epigenetic enzymes and the outcomes of GVHD models and clinical patients. Future studies mapping epigenetic mechanisms of IFN regulations in allogeneic T cells may be beneficial to elucidate how IFN modulates GVHD and GVL.

## Author Contributions

CZ conceived the manuscript and drew the figures. YZ and HZ discussed the concepts and critically revised the article. All authors contributed to the article and approved the submitted version.

## Conflict of Interest

The authors declare that the research was conducted in the absence of any commercial or financial relationships that could be construed as a potential conflict of interest.
